# Supplementary material for: Number Needed to Vaccinate with a Novel Tuberculosis Vaccine to Prevent Tuberculosis in High-Risk Populations, United States
Source: medRxiv. 2026 May 15:2026.05.11.26352950. Preprint. [Version 1] doi: 10.64898/2026.05.11.26352950 (PMC13193084; doi:10.64898/2026.05.11.26352950)
Supplement: Supplementary file 1 [file media-1.pdf]

Technical Appendix

Number Needed to Vaccinate with a Novel Tuberculosis Vaccine to Prevent Tuberculosis in High-Risk Populations, United States

Authors: Jessica E. Rothman, Kenneth G. Castro, Benjamin A. Lopman, Neel R. Gandhi, Kristin N. Nelson

Data Sources

Tuberculosis cases. We obtained 2023 TB case counts and the proportion of cases that can be attributed to reactivation (reactivation proportions) for U.S. high-risk groups from the National Tuberculosis Surveillance System (NTSS). *Mtb* infection prevalence estimates were averaged over 2018–2021 to adjust for COVID-19–related disruptions in TB surveillance during 2020–2021.

Reactivation rates. We used risk group-specific reactivation rates (rate of progression from *Mtb* infection to TB disease) and corresponding 95% confidence intervals (CIs) from 2011–2012 NHANES infection data measuring interferon-gamma release assay (IGRA) positivity (*1*).

Population denominators. Population size estimates in 2023 were obtained from the NIDDK US Renal Data System (USRDS) for ESRD (*2*), NCHHSTP AtlasPlus for HIV (*3*), US Diabetes Surveillance System for diabetes (*4*), and the American Community Survey (ACS) for nativity (*5*) (Appendix Table 1).

Vaccine efficacy. For this analysis, we used vaccine efficacy estimates from the M72/AS01$_E$ Phase IIb clinical trial, which demonstrated 49.7% efficacy (95% CI 2.1%–74.2%) in preventing progression to TB at 3 years post-vaccination among adolescents and adults in Kenya, South Africa, and Zambia with *Mtb* infection confirmed by IGRA positivity (*6*). We rounded this to 50% vaccine efficacy (VE) for our analysis.

Estimating *Mtb* Infection Burden

We adapted previously published back-calculation methodology (*7,8*) to estimate the number of individuals with *Mtb* infection within each risk group. This approach divides the number of TB cases attributed to reactivation by the corresponding reactivation rate.

After initial *Mtb* infection, individuals may progress to TB through two pathways: recent transmission (progression within 2 years of infection) or reactivation (progression after years of latent infection). The CDC uses the plausible source case method, which combines genotypic and epidemiological data to classify TB cases as resulting from either recent transmission or reactivation (*9*). From NTSS, we obtained TB case data stratified by risk group and the proportion of TB cases attributed to reactivation, as determined by the plausible source case method (*9,10*). We then applied previously published risk-group-specific reactivation rates (*1*) to calculate the number of *Mtb* infections in each group, i:

$$M_i = \frac{\text{Reactivation TB cases}_i}{\text{Reactivation rate}_i} \tag{1}$$

NNV Calculations

We calculated NNV under the two vaccination scenarios described in the main text, with the universal estimate serving as a reference. For all-comers vaccination (vaccinating all individuals within each risk group regardless of *Mtb* infection status):

$$\text{NNV}_i^{\text{all-comers}} = \frac{N_i}{t_i \times v} \tag{2}$$

For targeted vaccination (vaccinating only those with confirmed *Mtb* infection within each risk group):

$$\text{NNV}_i^{\text{targeted}} = \frac{M_i}{t_i \times v} \tag{3}$$

In Equations 1–3, i denotes the specific risk group, t_i is the number of TB cases, N_i is the population size, M_i is the number of *Mtb* infections estimated via Equation 1, and v is the vaccine efficacy estimate.

Bootstrap Confidence Intervals

We constructed 95% confidence intervals for the NNV estimates using parametric bootstrapping (1,000,000 iterations). For all-comers vaccination, population size was treated as fixed, with uncertainty in NNV derived from the number of TB cases (modeled using a Poisson distribution) and vaccine efficacy (modeled using a Beta distribution parameterized from the M72/AS01$_{\mathrm{E}}$ trial confidence interval). For targeted vaccination, the estimated number of *Mtb* infections was additionally treated as a random variable (lognormal distribution) to account for uncertainty in the back-calculation.

Appendix Table 1. Input parameters by risk group for NNV calculations.

Risk Group	Population Size (2023)	TB Cases (2023)	*Mtb* Infections (2018–21 avg)	*Mtb* Prev. (%)	TB Incidence (per 100k)
**Total U.S. population**	336,806,231	9,633	10,722,811	3.18	2.9
**By nativity**					
Non-U.S.-born	47,831,411	7,299	7,828,550	16.37	15.3
U.S.-born	287,083,485	2,292	2,831,426	0.99	0.8
**By medical condition**					
HIV	1,106,182	410	44,192	3.99	37.1
ESRD	803,055	254	46,595	5.80	31.6
Diabetes	31,733,668	2,251	2,227,132	7.02	7.1

Population sizes from: U.S. Census Bureau American Community Survey (nativity), CDC AtlasPlus (HIV), USRDS (ESRD), U.S. Diabetes Surveillance System (diabetes). TB cases from NTSS 2023. *Mtb* infection estimates from back-calculation, averaged over 2018–2021 to smooth COVID-19–related disruptions in TB surveillance during 2020–2021. *Mtb* prevalence = *Mtb* infections / population size × 100.

Appendix Table 2. Number needed to vaccinate to prevent one tuberculosis case by risk group, across vaccine efficacy assumptions of 40%–80%, in U.S. populations*

Risk Group	VE = 40%	VE = 50%	VE = 60%	VE = 70%	VE = 80%
All-comers vaccination					
Total U.S. population	87,409 (85,700–89,200)	69,928 (68,600–71,300)	58,273 (57,100–59,500)	49,948 (49,000–51,000)	43,705 (42,800–44,600)
Non-U.S.-born	16,383 (16,000–16,800)	13,106 (12,800–13,400)	10,922 (10,700–11,200)	9,362 (9,200–9,600)	8,191 (8,000–8,400)
U.S.-born	313,136 (301,000–326,000)	250,509 (241,000–261,000)	208,758 (201,000–218,000)	178,935 (172,000–187,000)	156,568 (150,000–163,000)
HIV	6,745 (6,100–7,500)	5,396 (4,900–6,000)	4,497 (4,100–5,000)	3,854 (3,500–4,300)	3,373 (3,100–3,700)
ESRD	7,904 (7,000–9,000)	6,323 (5,600–7,200)	5,269 (4,700–6,000)	4,517 (4,000–5,100)	3,952 (3,500–4,500)
Diabetes	35,244 (33,800–36,700)	28,195 (27,100–29,400)	23,496 (22,600–24,500)	20,139 (19,300–21,000)	17,622 (16,900–18,400)
Targeted vaccination					
Total U.S. population	2,783 (1,800–4,200)	2,226 (1,400–3,300)	1,855 (1,200–2,800)	1,590 (1,000–2,400)	1,391 (880–2,100)
Non-U.S.-born	2,681 (2,100–3,400)	2,145 (1,700–2,700)	1,788 (1,400–2,300)	1,532 (1,200–1,900)	1,341 (1,000–1,700)
U.S.-born	3,088 (950–7,600)	2,471 (750–6,100)	2,059 (630–5,100)	1,765 (540–4,300)	1,544 (470–3,800)
HIV	269 (150–450)	216 (120–360)	180 (100–300)	154 (86–260)	135 (75–220)
ESRD	459 (200–910)	367 (160–730)	306 (130–610)	262 (110–520)	229 (100–460)
Diabetes	2,473 (1,600–3,700)	1,979 (1,200–3,000)	1,649 (1,000–2,500)	1,413 (890–2,100)	1,237 (780–1,900)

*NNV, number needed to vaccinate; VE, vaccine efficacy; ESRD, end-stage renal disease; *Mtb*, *Mycobacterium tuberculosis*. Cells display NNV point estimate (95% CI). NNV estimates were generated by holding vaccine efficacy fixed at the indicated value while sampling TB case counts and the *Mtb*-infected subpopulation size from their respective bootstrap distributions (1,000,000 iterations). All-comers vaccination assumes vaccination of all individuals in the risk group regardless of *Mtb* infection status; targeted vaccination restricts vaccination to those with confirmed *Mtb* infection.

Appendix Table 3. Number needed to vaccinate (NNV) to prevent one outcome of interest, comparison of an M72/AS01$_E$-like tuberculosis vaccine to other established vaccines*

**Vaccine**	**Outcome**	**Population**	**NNV (95% CI)**	**Reference**
Tuberculosis (this study, targeted vaccination)				
M72/AS01$_E$	TB disease prevention	PLWH, *Mtb*-infected	217 (95–755)	This study
M72/AS01$_E$	TB disease prevention	ESRD, *Mtb*-infected	369 (133–1,370)	This study
M72/AS01$_E$	TB disease prevention	Diabetes, *Mtb*-infected	1,991 (960–6,800)	This study
M72/AS01$_E$	TB disease prevention	Non-U.S.-born, *Mtb*-infected	2,158 (1,200–7,100)	This study
M72/AS01$_E$	TB disease prevention	U.S.-born, *Mtb*-infected	2,486 (660–10,100)	This study
Tuberculosis (this study, all-comers vaccination)				
M72/AS01$_E$	TB disease prevention	PLWH, all	5,429 (3,200–17,800)	This study
M72/AS01$_E$	TB disease prevention	ESRD, all	6,361 (3,700–20,800)	This study
M72/AS01$_E$	TB disease prevention	Non-U.S.-born, all	13,185 (7,800–42,700)	This study
M72/AS01$_E$	TB disease prevention	U.S.-born, all	252,021 (148,500–824,000)	This study
Tuberculosis (modeled, Harris et al. 2020, China)				
TB vaccine (modeled)	TB disease prevention	60–64y, pre-infection only	1,022	(*11*)
TB vaccine (modeled)	TB disease prevention	60–64y, post-infection latency	576	(*11*)
TB vaccine (modeled)	TB disease prevention	60–64y, post-infection L&R	281	(*11*)
TB vaccine (modeled)	TB disease prevention	60–64y, pre- & post-infection	230	(*11*)
Established adult vaccines				
Shingrix	Herpes zoster	Adults ≥50y	12 (10–14)	(*12*)
Influenza	Influenza illness	Adults ≥65y (Australia)	40 (15–250)	(*13*)
COVID-19 booster	Hospitalization	Adults	205 (44–615)	(*14*)
PCV-13	All CAP	Adults ≥65y (5y)	234	(*15*)
Zostavax	HZ hospitalization	Adults ≥65y	263 (210–340)	(*16*)
PCV-13	Hospital CAP	Adults ≥65y (5y)	537	(*15*)
HPV	Cervical cancer mortality	Girls vaccinated at 12y	324	(*17*)
HPV	Cervical cancer death	Adult women	835	(*17*)
Influenza	Hospitalization	Adults ≥65y	850 (520–1,800)	(*18*)
PCV-13	Outpatient CAP	Adults ≥65y (1y)	1,287	(*15*)
PCV-13	Hospital CAP	Adults ≥65y (1y)	1,620	(*15*)
Influenza	Death	Adults ≥65y (Australia)	5,000 (1,400–20,000)	(*13*)

*NNV, number needed to vaccinate; CI, confidence interval; PLWH, persons living with HIV; ESRD, end-stage renal disease; *Mtb*, *Mycobacterium tuberculosis*; PCV-13, 13-valent pneumococcal conjugate vaccine; HPV, human papillomavirus; HZ, herpes zoster; CAP, community-acquired pneumonia; L&R, latent and recovered. NNV estimates from this study assume 50% vaccine efficacy and a 3-year time horizon. Comparator NNV values reflect universal or age-targeted vaccination programs and are shown for context; direct comparison with risk-targeted estimates from this study is limited by differences in target population and study design.

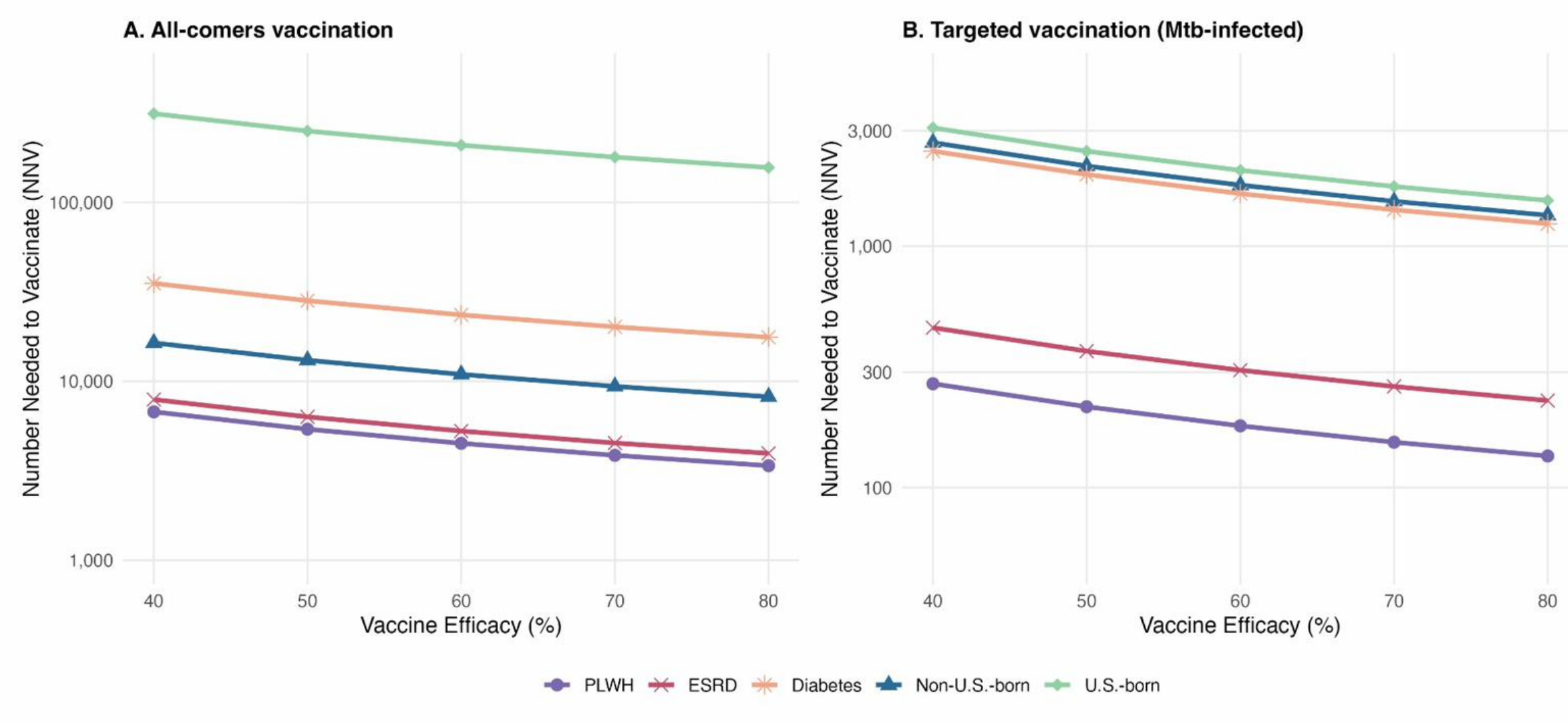

Appendix Figure 1. Number needed to vaccinate (NNV) by risk group across vaccine efficacy assumptions of 40%, 50%, 60%, 70%, and 80% in U.S. populations. Panel A shows NNV under all-comers vaccination (vaccinating all individuals in a risk group regardless of *Mtb* infection status); Panel B shows NNV under targeted vaccination (vaccinating only those with confirmed *Mtb* infection). NNV is plotted on a log scale. PLWH, persons living with HIV; ESRD, end-stage renal disease.

Appendix References

1. Ekramnia M, Li Y, Haddad MB, Marks SM, Kammerer JS, Swartwood NA, et al. Estimated rates of progression to tuberculosis disease for persons infected with *Mycobacterium tuberculosis* in the United States. Epidemiology. 2024;35:164–73.

2. National Institute of Diabetes and Digestive and Kidney Diseases. ESRD prevalent count—USRDS. Bethesda (MD): NIDDK; 2024 [cited 2024 May 4]. https://www.niddk.nih.gov/about-niddk/strategic-plans-reports/usrds/data-query-tools/esrd-prevalent-count

3. Centers for Disease Control and Prevention. NCHHSTP AtlasPlus—tables. Atlanta: CDC; 2024 [cited 2025 Oct 20]. https://gis.cdc.gov/grasp/nchhstpatlas/tables.html

4. Centers for Disease Control and Prevention. United States Diabetes Surveillance System. Atlanta: CDC; 2024 [cited 2025 Oct 20]. https://gis.cdc.gov/grasp/diabetes/diabetesatlas-surveillance.html

5. U.S. Census Bureau. American Community Survey (ACS). Washington: U.S. Census Bureau; 2024 [cited 2024 May 4]. https://www.census.gov/programs-surveys/acs

6. Tait DR, Hatherill M, Van Der Meeren O, Ginsberg AM, Van Brakel E, Salaun B, et al. Final analysis of a trial of M72/$AS01_E$ vaccine to prevent tuberculosis. N Engl J Med. 2019;381:2429–39.

7. Haddad MB, Raz KM, Lash TL, Hill AN, Kammerer JS, Winston CA, et al. Simple estimates for local prevalence of latent tuberculosis infection, United States, 2011–2015. Emerg Infect Dis. 2018;24:1930–3.

8. Mirzazadeh A, Kahn JG, Haddad MB, Hill AN, Marks SM, Readhead A, et al. State-level prevalence estimates of latent tuberculosis infection in the United States by medical risk factors, demographic characteristics and nativity. PLOS ONE. 2021;16:e0249012.

9. France AM, Grant J, Kammerer JS, Navin TR. A field-validated approach using surveillance and genotyping data to estimate tuberculosis attributable to recent transmission in the United States. Am J Epidemiol. 2015;182:799–807.

10. CDCTB. Reported TB in the U.S., 2021—Estimates of recent transmission. Atlanta: Centers for Disease Control and Prevention; 2022 [cited 2023 Apr 3]. https://www.cdc.gov/tb/statistics/reports/2021/estimates.htm

11. Harris RC, Sumner T, Knight GM, Zhang H, White RG. Potential impact of tuberculosis vaccines in China, South Africa, and India. Sci Transl Med. 2020;12:eaax4607.

12. Lal H, Cunningham AL, Godeaux O, Chlibek R, Diez-Domingo J, Hwang SJ, et al. Efficacy of an adjuvanted herpes zoster subunit vaccine in older adults. N Engl J Med. 2015;372:2087–96.

13. Kelly H, Attia J, Andrews R, Heller RF. The number needed to vaccinate (NNV) and population extensions of the NNV: comparison of influenza and pneumococcal vaccine programmes for people aged 65 years and over. Vaccine. 2004;22:2192–8.

14. Graciaa DS, Schmidt MA, Naleway AL, Goddard K, Crane B, Vesco KK, et al. Number needed to vaccinate with a COVID-19 booster to prevent a COVID-19-associated hospitalization during SARS-CoV-2 Omicron BA.1 variant predominance, December 2021–February 2022, VISION Network: a retrospective cohort study. Lancet Reg Health Am. 2023;23:100530.

15. McLaughlin JM, Jiang Q, Isturiz RE, Sings HL, Swerdlow DL, Gessner BD, et al. Effectiveness of 13-valent pneumococcal conjugate vaccine against hospitalization for community-acquired pneumonia in older US adults: a test-negative design. Clin Infect Dis. 2018;67:1498–506.

16. Oxman MN, Levin MJ, Johnson GR, Schmader KE, Straus SE, Gelb LD, et al. A vaccine to prevent herpes zoster and postherpetic neuralgia in older adults. N Engl J Med. 2005;352:2271–84.

17. Brisson M, Van de Velde N, De Wals P, Boily MC. The potential cost-effectiveness of prophylactic human papillomavirus vaccines in Canada. Vaccine. 2007;25:5399–408.

18. Chung JR, Rolfes MA, Flannery B, Prasad P, O'Halloran A, Garg S, et al. Effects of influenza vaccination in the United States during the 2018–2019 influenza season. Clin Infect Dis. 2020;71:e368–76.
